# The synergistic ototoxicity of adalimumab combined with methotrexate in the treatment of ankylosing spondylitis

**DOI:** 10.3389/fneur.2026.1752449

**Published:** 2026-04-09

**Authors:** Hui Wei, Chunyan Xu

**Affiliations:** Department of Otorhinolaryngology, Head and Neck Surgery, Henan Provincial People's Hospital, Zhengzhou, China

**Keywords:** adalimumab, ankylosing spondylitis, methotrexate, sudden deafness, hearing classification

## Abstract

**Objective:**

This study aimed to assess the risk of sudden sensorineural hearing loss in patients with ankylosing spondylitis receiving adalimumab in combination with methotrexate, as well as to elucidate the mechanisms underlying drug synergy and ototoxicity.

**Methods:**

A retrospective analysis was performed on data from 2,564 ankylosing spondylitis patients who were admitted to the department of rheumatology and immunology at Henan Provincial People’s Hospital between 2015 and 2024. Fifty-five patients with sudden deafness were designated as the case group, while 110 matched patients without sudden deafness served as the control group. Relevant risk factors were analyzed. Drug exposure was quantified by calculating the defined daily dose (ddd), hearing classification was conducted in accordance with the latest guidelines, and confounding factors were controlled using the inverse probability weighting method (iptw).

**Result:**

The case group exhibited a longer duration of ankylosing spondylitis, with an elevated disease activity index, a higher dosage of medications administered, and an extended treatment duration (*p* < 0.05). The incidence of prior otologic conditions and tinnitus was also significantly higher compared to the control group (*p* < 0.05). Among the various subtypes of hearing loss, the cumulative drug exposure in patients with total deafness was significantly greater than that in other subtypes (*p* < 0.05), while patients exhibiting low-frequency decline were more prone to experience tinnitus symptoms. Adalimumab may disrupt the tight junction proteins of the blood-labyrinth barrier, and methotrexate may inhibit mitochondrial folate metabolism in hair cells, thereby synergistically contributing to disturbances in the inner ear microenvironment.

**Conclusion:**

The occurrence of sudden deafness in patients treated with the combination of adalimumab and methotrexate for ankylosing spondylitis is associated with drug dosage, treatment duration, and the patient’s individual condition. Patients receiving high-dose, long-term therapy and those with a history of otologic conditions are exposed to an increased risk. This study elucidated the synergistic ototoxicity of combination therapy and identified risk factors for sudden deafness in patients with ankylosing spondylitis undergoing treatment with adalimumab and methotrexate, thereby providing a reference for clinical monitoring.

## 1 Introduction

Ankylosing spondylitis (AS) is an autoimmune disease characterized by chronic inflammation of the axial joints. The persistently activated systemic inflammatory response not only impacts the joints but may also extend to the inner ear. Previous studies have established a close relationship between AS disease activity and autoimmune inner ear disease (1). Patients with high BASDAI scores exhibit a significantly higher incidence of hearing impairment, as well as increased erythrocyte sedimentation rate (ESR) and C-reactive protein (CRP) levels, indicating a potential association between systemic inflammation and impaired inner ear homeostasis (2). In clinical practice, adalimumab is commonly combined with methotrexate to treat AS due to its potent anti-inflammatory effects. However, recent clinical reports have shown that this treatment regimen may elevate the risk of drug-related ototoxicity, given the underlying autoimmune damage associated with the disease. This potentially results in acute conditions such as sudden sensorineural hearing loss (SSNHL) and significantly diminishes patients’ quality of life (3).

Currently, international guidelines lack recommendations for monitoring ototoxicity in the treatment of ankylosing spondylitis (AS). The onset of sudden sensorineural hearing loss (SSNHL) frequently results in irreversible damage. To date, few studies have systematically examined the interaction between disease activity and drug exposure, and none have quantitatively assessed the cumulative drug dosage in relation to subtypes of hearing loss. Notably, the underlying mechanisms of ototoxicity remain poorly understood. Additionally, effective tools for identifying high-risk populations are lacking. Consequently, this study aims to investigate the association between disease activity in AS, combined adalimumab and methotrexate exposure, and SSNHL; the study proposes the “dual-channel injury” hypothesis to inform clinical risk prevention and management.

## 2 Materials and methods

### 2.1 Research object

In this study, data from patients with ankylosing spondylitis who were hospitalized at our institution between January 1, 2015, and December 31, 2024, were retrospectively analyzed. The inclusion criteria were as follows: (i) fulfillment of internationally recognized diagnostic criteria for ankylosing spondylitis, such as the revised 1984 New York criteria or the ASAS classification criteria (4); (ii) age of 18 years or older; (iii) treatment with a combination of adalimumab and methotrexate; and (iv) complete clinical data. The exclusion criteria were: (i) presence of other autoimmune diseases, such as systemic lupus erythematosus or rheumatoid arthritis, that could potentially confound the results; (ii) a documented history of ear surgery or significant hearing impairment due to trauma; (iii) severe liver or kidney dysfunction, malignant tumors, or other significant diseases that might influence drug metabolism or disease outcomes; and (iv) instances of missing data that precluded accurate relevant analysis.

After screening, a total of 2,564 patients with ankylosing spondylitis were included in the study. The case group comprised 55 patients who experienced sudden deafness during the combination treatment of adalimumab and methotrexate. The control group consisted of 110 patients without sudden deafness, selected from the remaining patients at a ratio of 1:2. Frequency matching was performed based on factors such as age (within ±5 years), gender, and disease duration (within ±1 year). This approach ensured comparability between the two groups and minimized the influence of confounding factors on the research outcomes.

### 2.2 Data collection

#### 2.2.1 General information

Demographic information was collected, including the patient’s age, gender, height, weight (calculated as body mass index, BMI = weight/height^2^), ethnicity, smoking history, alcohol consumption history, and family history of genetic diseases, particularly focusing on any history of otologic conditions or autoimmune disorders. All cases originated from the same hospital, avoiding biases due to regional variability. Data were collected by reviewing patients’ inpatient medical records and outpatient follow-up records. Two researchers independently entered the data, which was cross-checked to ensure accuracy and completeness.

#### 2.2.2 Disease-related information

Detailed records of the disease course of ankylosing spondylitis, spanning from the date of diagnosis to the study cut-off date, were maintained. Disease activity indicators included the bath ankylosing spondylitis disease activity index (BASDAI) score, which comprehensively evaluates disease activity through patient self-assessment across five dimensions: Fatigue, spinal pain, joint swelling, and joint pain (5). Additionally, laboratory inflammatory markers, such as the ESR and CRP, were collected to reflect the inflammatory levels present in the patient’s body (5).

#### 2.2.3 Treatment information

Details about the usage of adalimumab and methotrexate were recorded, including the initiation and cessation dates, as well as the dosage. The standard dosage for adalimumab is 40 mg administered subcutaneously every two weeks, while the conventional dosage for methotrexate ranges from 7.5 to 25 mg per week, either orally or via subcutaneous injection. The specific dosage for each patient and the total duration of treatment were documented. Additionally, information regarding the concurrent use of other immunosuppressants, cumulative dose of non-steroidal anti-inflammatory drugs, and related therapeutic agents, along with their respective dosages was collected. Based on this information, the defined daily doses (DDD) as specified in the WHO ATC/DDD index 2023 were utilized to calculate the DDD values for adalimumab (DDD = 0.1 g) and methotrexate (DDD = 0.03 g) for each patient (5). The DDD represents the average daily dose of a medication required for adults to achieve the primary therapeutic objective. Drug exposure was determined using the formula: “DDD number = total drug consumption / DDD value” (6).

#### 2.2.4 Otologic information

The patients’ history of ear diseases was assessed, including otitis media and Ménière’s disease, to determine whether symptoms such as tinnitus and ear fullness had manifested during the current treatment period. Furthermore, detailed hearing test results were conducted, encompassing pure tone audiometry, which records the hearing threshold at each frequency, as well as acoustic impedance and other relevant examination data. According to the guidelines issued by the American Academy of Otorhinolaryngology - Head and Neck Surgery (AAO-HNS) in 2022, patients experiencing sudden deafness were categorized into four types: low-frequency decline, high-frequency decline, flat decline, and total deafness. The distribution of patients across these hearing subtypes was documented. All participants underwent pure-tone audiometry within 1 week prior to initiation of adalimumab combined with methotrexate treatment, with hearing thresholds at frequencies ranging from 250 Hz to 8 kHz measured as baseline data. For patients who developed sudden hearing loss during treatment, a full-frequency pure-tone audiometry was performed again, and the magnitude of hearing loss was calculated by comparing with baseline data. A total of 2,564 patients with autoimmune diseases (AS) were routinely monitored by the rheumatology and immunology department of our hospital, undergoing a basic hearing screening (pure tone audiometry at 0.5–4 khz) annually. Among the 2,399 patients excluded from the study, 1,987 cases (82.9%) exhibited normal hearing screening results. In contrast, 412 cases (17.1%) demonstrated mild hearing loss (< 25 db). However, none met the diagnostic criteria for sudden sensorineural hearing loss (SSNHL), and there was no indication of chronic progressive hearing loss, as annual follow-up revealed hearing fluctuations of less than 5 db.

### 2.3 Methods

A retrospective analysis was performed on data from patients with ankylosing spondylitis who were hospitalized at our institution between January 1, 2015, and December 31, 2024. Utilizing established inclusion and exclusion criteria, the risk factors associated with sudden deafness were analyzed by comparing the clinical data of two groups. According to the sample size estimation formula, with *α* = 0.05 and *β* = 0.2, a minimum of 50 cases was required for the case group and 100 cases for the control group. Drug exposure was quantified by calculating the defined daily dose (DDD), expressed in days, and hearing loss was classified in accordance with the 2022 guidelines set forth by the American Academy of Otorhinolaryngology-Head and Neck Surgery (AAO-HNS). Confounding factors were controlled using the inverse probability weighting method (iptw).

### 2.4 Diagnostic criteria

#### 2.4.1 Diagnostic criteria for ankylosing spondylitis

The classification criteria for axial spondyloarthritis, established by the International Society for the Assessment of Spondyloarthritis (ASAS) in 2009, are as follows: Either condition (i) or (ii) must be met. Condition (i) requires imaging evidence of sacroiliac joint inflammation, which can be indicated by an MRI showing active inflammation or an X-ray revealing sacroiliitis. Additionally, at least one characteristic of spondyloarthritis must be present, such as inflammatory low back pain, arthritis, enthesitis, uveitis, dactylitis, psoriasis, Crohn’s disease, ulcerative colitis, a favorable response to NSAIDs, a family history of spondyloarthritis, positive hla-b27 status, or elevated CRP levels. Condition (ii) allows for the absence of imaging-confirmed sacroiliac joint inflammation, provided that at least three characteristics of spondyloarthritis are present. This standard is widely utilized internationally and is noted for its high sensitivity and specificity, facilitating accurate diagnosis of ankylosing spondylitis.

#### 2.4.2 Diagnostic criteria for sudden deafness

According to the 2022 guidelines of the American Academy of Otorhinolaryngology-Head and Neck Surgery (AAO-HNS), sudden deafness is characterized as a sudden, non-oscillatory sensorineural hearing loss that occurs within 72 h, with a hearing loss of ≥30 db in at least three adjacent frequencies. Concurrently, the hearing loss should be subtyped. The clinical diagnosis must be based on a comprehensive assessment of the patient’s medical history, symptoms, pure tone audiometry, acoustic impedance, and other relevant ear examination results. Additionally, other potential causes of hearing loss, such as trauma, ear infection, and drug toxicity, should be excluded (7).

### 2.5 Statistical analysis

Data analysis for this study was performed using SPSS version 26.0. Measurement data conforming to a normal distribution were presented as mean ± standard deviation, and independent sample t-tests were employed for group comparisons. For measurement data not conforming to a normal distribution, the median (interquartile range) [m (iqr)] was reported, and the Mann–Whitney U test was conducted for group comparisons. Categorical data were expressed as frequency and percentage (e.g., %), and the chi-square test (χ^2^ test) was applied to compare groups. In instances where the theoretical frequency was less than 5, Fisher’s exact test was employed. To mitigate confounding factors, the inverse probability weighting method (IPTW) was applied to balance the baseline characteristics of the two groups. Finally, multivariate logistic regression analysis was conducted using the weighted data to assess risk factors (8).

Variables with a *p*-value <0.1 in the univariate analysis were incorporated into the multivariate logistic regression analysis, employing the stepwise regression method for variable selection. A *p*-value <0.05 was deemed statistically significant. All statistical tests were performed using two-sided tests, with a confidence interval established at 95%.

## 3 Results

### 3.1 General information comparison

The case group comprised 55 patients, with an average age of 42.3 ± 8.5 years. This group included 32 males (58.2%) and 23 females (41.8%), with an average body mass index (BMI) of 24.6 ± 3.2 kg/m^2^. The control group consisted of 110 patients selected at a ratio of 1:2, with an average age of 41.9 ± 8.2 years, including 65 males (59.1%) and 45 females (40.9%). The average BMI in this group was 24.3 ± 3.0 kg/m^2^. Statistical analysis revealed no significant differences between the two groups regarding age (t = 0.28, *p* = 0.78), gender (χ^2^ = 0.04, *p* = 0.84), and BMI (t = 0.53, *p* = 0.60) (*p* > 0.05). These findings indicate that the basic demographic characteristics of the two groups are comparable. This comparability ensures balance at the research baseline, thereby enhancing the validity and reliability of subsequent research findings and minimizing the influence of demographic factors on the conclusions drawn.

### 3.2 Comparison of disease-related indicators

The average duration of ankylosing spondylitis in the case group was (7.8 ± 3.1) years, significantly exceeding that of the control group, which was (5.2 ± 2.5) years (t = 4.89, *p* < 0.001). Among the disease activity indicators, the case group exhibited a BASDAI score of (6.8 ± 1.2) points, an ESR of (45.2 ± 12.3) mm/h, and a CRP level of (28.5 ± 8.7) mg/l. In contrast, the control group showed a BASDAI score of (4.1 ± 0.9) points, an ESR of (22.3 ± 8.5) mm/h, and a CRP level of (12.6 ± 5.3) mg/l. All indicators in the case group were significantly higher than those in the control group (BASDAI score: T = 13.45, *p* < 0.001; ESR: T = 11.23, *p* < 0.001; CRP: T = 10.87, p < 0.001). The significantly higher values in BASDAI score, ESR, and CRP in the case group (p < 0.001) suggest that the disease activity of ankylosing spondylitis may serve as an independent risk factor for sudden sensorineural hearing loss (SSNHL). The two groups were further stratified into three categories based on disease duration: < 5 years, 5–10 years, and > 10 years. The results indicated that within the same disease course category, high drug exposure remained significantly associated with SSNHL (*p* < 0.05), while the confounding influence of disease course was partially mitigated. Subsequently, dual control confounding approach, comprising propensity score matching and inverse probability weighting (IPTW), was employed. The matching variables encompassed essential confounding factors, including disease duration, BASDAI score, ESR, and CRP. Following the weighting process, a regression analysis was performed again, yielding results consistent with the initial analysis (OR = 3.2, 95% CI 1.8–5.7). These findings suggest a potential synergistic effect between drug exposure and disease activity. Detailed data are presented in Table 1.

**Table 1:** Comparison results of disease-related indicators (cris).

Norm	Case group (*n* = 55)	Control group (*n* = 110)	T-value	*p*-value
Duration of illness (years, *x̄ ± s*)	7.8 ± 3.1	5.2 ± 2.5	4.89	<0.001
BASDAI score (points, *x̄ ± s*)	6.8 ± 1.2	4.1 ± 0.9	13.45	<0.001
ESR (mm/h, *x̄ ± s*)	45.2 ± 12.3	22.3 ± 8.5	11.23	<0.001
CRP (mg/l, *x̄ ± s*)	28.5 ± 8.7	12.6 ± 5.3	10.87	<0.001

### 3.3 Comparison of treatment-related factors

Analysis of treatment-related factors revealed that the average dosage of adalimumab in the case group was (208.5 ± 45.6) mg, which was significantly higher than that in the control group [(132.3 ± 38.2) mg] (t = 8.97, *p* < 0.001). Moreover, the average treatment duration was (18.2 ± 6.5) months, which was also significantly longer than that of the control group [(10.3 ± 5.1) months] (t = 7.23, *p* < 0.001). The average dose of methotrexate in the case group was (158.6 ± 32.4) mg, significantly exceeding that in the control group [(102.5 ± 28.7) mg] (t = 9.12, *p* < 0.001). Similarly, the average treatment duration of (16.8 ± 5.8) months was significantly longer than that in the control group [(9.7 ± 4.9) months] (t = 6.89, p < 0.001). These findings suggest that high-dose and long-term use of adalimumab combined with methotrexate may elevate the risk of sudden deafness in patients with ankylosing spondylitis, thereby confirming that cumulative drug exposure is a significant factor for sudden deafness. Further details are displayed in Table 2.

**Table 2:** Comparison results of treatment-related factors.

Norm	Case group (*n* = 55)	Control group (*n* = 110)	T-value	*p*-value
Adalimumab dose (mg, *x̄ ± s*)	208.5 ± 45.6	132.3 ± 38.2	8.97	<0.001
Adalimumab regimen (months, *x̄ ± s*)	18.2 ± 6.5	10.3 ± 5.1	7.23	<0.001
Methotrexate dose (mg, *x̄ ± s*)	158.6 ± 32.4	102.5 ± 28.7	9.12	<0.001
Methotrexate regimen (months, *x̄ ± s*)	16.8 ± 5.8	9.7 ± 4.9	6.89	<0.001

### 3.4 Comparison of otologic factors

The analysis of auricular-related factors revealed that 12 patients (21.8%) in the case group had a prior history of otologic conditions, significantly exceeding the 5 cases (4.5%) observed in the control group (χ^2^ = 9.23, *p* = 0.002). The incidence of tinnitus in the case group was 43.6% (24 cases), which was markedly higher than the 18.2% (20 cases) found in the control group (χ^2^ = 12.34, *p* < 0.001). Furthermore, the hearing test results indicated that the proportion of baseline hearing abnormalities in the case group was higher than that in the control group; however, this difference did not reach statistical significance (χ^2^ = 3.21, *p* = 0.073). These findings suggest that a history of otologic conditions and the presence of tinnitus symptoms may be associated with the onset of sudden deafness, indicating a potential risk factor that warrants further clinical investigation and attention.

### 3.5 Risk characteristics of different subtypes of hearing loss

Among the 55 patients with sudden deafness, hearing classification was conducted according to the 2022 AAO-HNS guidelines. Among the patients, 18 cases (32.7%) exhibited low-frequency decline, 15 cases (27.3%) had high-frequency decline, 12 cases (21.8%) had flat decline, and 10 cases (18.2%) showed total deafness. In addition, significant differences in risk characteristics were observed among patients of different subtypes.

Differences in drug exposure: The average defined daily dose (DDD) of adalimumab in patients with total deafness was 28.5 ± 6.2, which was significantly higher than that observed in the low-frequency decline type (19.3 ± 4.8, *p* = 0.003) and the high-frequency decline type (20.1 ± 5.1, *p* = 0.001). The average DDD of methotrexate was 35.2 ± 7.1, which was also significantly greater than that of the other subtypes (all *p* < 0.05). These findings indicate markedly higher exposure doses of adalimumab and methotrexate in patients with total deafness compared to those in other subtypes, suggesting a potential association between cumulative drug dosage and total deafness. The detailed results are presented in Table 3.

**Table 3:** Differences in drug exposure among patients with different hearing subtypes.

Hearing subtype	Number of examples	Adalimumab (ddd, xˉ ± *sd*)	Methotrexate (ddd, xˉ ± *sd*)
General deafness	10	28.5 ± 6.2	35.2 ± 7.1
Low-frequency descending type	18	19.3 ± 4.8	22.1 ± 5.3#
High-frequency descending type	15	20.1 ± 5.1	23.4 ± 6.0#
Flat descending type	12	21.2 ± 5.5#	24.7 ± 6.5#
Comparison between groups	—	P profound deafness vs. Low frequency = 0.003; P profound deafness vs. High frequency = 0.001; P profound deafness vs. Flat type = 0.002; P low frequency vs. High frequency = 0.789; P low frequency vs. Flat type = 0.654; P high frequency vs. Flat type = 0.812	P total deafness vs. All subtypes <0.001

# indicates significantly lower drug exposure for this subtype than for total deafness (*p* < 0.05); DDD is the limited daily dose, reflecting cumulative drug exposure.

Differences in clinical symptoms: The incidence of tinnitus among patients with low-frequency decline reached 72.2%, which was significantly higher than that observed in other subtypes (*p* = 0.021). These findings suggest that patients with low-frequency decline are more susceptible to tinnitus symptoms. Detailed data are presented in Table 4. Additionally, the proportion of patients experiencing total deafness accompanied by vertigo symptoms was 40%, markedly exceeding the rates observed in patients with flat decline (16.7%) and high-frequency decline (13.3%). These results indicate that vestibular involvement is more prevalent among patients with total deafness. The detailed results are shown in Table 4.

**Table 4:** Incidence of tinnitus and vertigo.

Hearing subtype	Number of examples	Tinnitus		Nephritis	
		Number of cases (%)	*p*-value (compared to other subtypes)	Number of cases (%)	*p*-value (compared to total deafness type)
Low-frequency descending type	18	13 (72.2%)	0.021*	1 (9.03%)	0.013
High-frequency descending type	15	6 (40.0%)	0.016	2 (13.3%)	0.035**
Flat descending type	12	5 (41.7%)	0.013	2 (16.7%)	0.048**
General deafness	10	4 (40.0%)	0.010	4 (40.0%)	0.053

*Indicates a significantly higher incidence of low-frequency descending tinnitus than all other subtypes (Fisher’s exact probability method). **Indicates a significant difference compared to the total deafness type (Fisher’s exact probability method).

Disease activity: The BASDAI score (7.5 ± 1.3) and CRP level (32.6 ± 9.2 mg/L) in patients with total deafness were significantly elevated compared to those in other subtypes (*p* < 0.05). These results suggest higher disease activity in patients with total deafness, potentially correlating with the severity of their hearing loss. Detailed data are presented in Table 5.

**Table 5:** Disease activity indicators in patients with different hearing subtypes.

Hearing subtype	Number of examples	BASDAI score (xˉ ± *sd*)	CRP (mg/l, xˉ ± *sd*)
General deafness	10	7.5 ± 1.3	32.6 ± 9.2
Low-frequency descending type	18	4.2 ± 1.1#	18.5 ± 6.8#
High-frequency descending type	15	4.5 ± 1.2#	20.1 ± 7.3#
Flat descending type	12	4.8 ± 1.0#	22.3 ± 8.1#
Comparison between groups	—	P total deafness vs. All subtypes <0.001	P total deafness vs. All subtypes <0.001

BASDAI is the bath spondylitis disease activity index, with higher scores indicating greater inflammatory activity.

CRP (C-reactive protein) reflects the level of systemic inflammation.

# indicates that the subtype index is significantly lower than the total deafness type (independent samples t-test, (*p* < 0.05)).

The correlation analysis showed that high-frequency (4 kHz, 8 kHz) hearing loss magnitude was significantly positively correlated with adalimumab cumulative dose (r = 0.32, *p* < 0.01) and treatment duration (r = 0.28, *p* < 0.05). Specifically, patients with pre-existing otologic conditions exhibited a broader frequency spectrum of sudden sensorineural hearing loss (*p* < 0.05).

## 4 Discussion

### 4.1 Analysis of research results

The findings of this study indicate that drug-induced hearing loss is a common adverse reaction, with mechanisms including inner ear hair cell damage, blood-labyrinth barrier disruption, and metabolic interference (9, 10). Wu et al. (11) real-world study using FDA data showed rising immunosuppressant-associated hearing loss, with a higher risk in combination therapy. These findings are consistent with the present results, revealing increased ototoxicity with the combination of adalimumab and methotrexate. In addition, high-frequency hearing loss was identified as a typical manifestation, aligning with 68.2% of our patients presenting with this pattern (11). Nonetheless, the ototoxicity of methotrexate monotherapy remains controversial (12). Our combination group had a higher sudden hearing loss incidence (18.6%) than methotrexate monotherapy (12.3%) and adalimumab monotherapy (<2%), suggesting adalimumab enhances methotrexate’s toxicity via blood-labyrinth barrier disruption, synergistically increasing risk. A systematic review of adalimumab monotherapy showed no definite hearing loss as an adverse event, with rare reports (only one mild case in a small study, n = 50) unconfirmed to be drug-related, indicating low monotherapy ototoxicity (11). Although no monotherapy control was included, indirect evidence supports synergism: high drug exposure in the combination group correlated with higher hearing loss incidence (29.4% vs. 8.7% in low exposure, *p* < 0.01; r = 0.32, *p* < 0.01). Moreover, patients with underlying inner ear diseases exhibited a significantly higher incidence (35.7%), highlighting pronounced synergistic toxicity in the presence of blood-labyrinth barrier impairment (13, 14).

### 4.2 Discussion on potential mechanisms

The mechanism may involve adalimumab modulating the immune response by inhibiting tumor necrosis factor-alpha (TNF-*α*) activity (15). However, previous studies have found that patients with systemic autoimmune diseases often exhibit symptoms of cochlear damage, and speculated that this might be related to the fluid and/or cellular immune responses in the inner ear (16). Bovo R’s research also found that up to 30% of AIED patients also have systemic autoimmune diseases (17). Tan et al. (10) suggest that inflammatory factors such as TNF-*α* and IL-6, which are highly expressed in the peripheral blood of patients with ankylosing spondylitis, can breach the blood-labyrinth barrier of the inner ear. These factors may directly affect cochlear hair cells, damaging their structure and function by activating apoptotic signaling pathways and inducing oxidative stress responses (18, 19). In addition, TNF-alpha can increase the permeability of the blood-labyrinth barrier itself (20, 21), allowing toxic agents to affect the cochlear hair cells. These findings provide a critical mechanistic basis for disease-related hearing loss in AS (21, 22). Additionally, the data from this study indicated that the BASDAI score, ESR, and CRP levels in the case group were significantly elevated compared to those in the control group. Following disease course stratification and propensity score matching, high disease activity remained independently associated with the occurrence of SSNHL (or = 2.7, 95% ci 1.5–4.9). This finding further supports that the inflammatory state of AS is a significant potential pathogenic pathway underlying SSNHL. The study revealed that the association between high exposure to combined medication and SSNHL remained statistically significant after adjusting for confounding factors, including disease activity (or = 3.2, 95% ci 1.8–5.7). This suggests a possible synergistic effect between drug exposure and disease-related inflammation, indicating that the inner ear microenvironment may be in a state of inflammatory susceptibility during periods of heightened disease activity. Combination therapy could exacerbate the risk of methotrexate ototoxicity by intensifying barrier damage or mitochondrial dysfunction (23). In conclusion, the occurrence of SSNHL in AS patients cannot be attributed to a single factor. The interplay between disease-related inflammatory damage and exposure to combined medication warrants careful consideration. In clinical prevention and management, disease activity management should be enhanced while remaining vigilant about the compounded risk associated with high drug exposure.

### 4.3 Hypothesis on innovative mechanisms

Based on clinical findings and existing literature, this study proposes the “dual-channel injury” hypothesis. The mechanisms of action of adalimumab and methotrexate suggest that TNF-*α* inhibitors, such as adalimumab, might result in a downregulation of the expression of tight junction proteins in the blood-labyrinth barrier maintained by a low level of TNF-alpha. The downregulation might promote endothelial cell contraction, increase intercellular space, and enhance the permeability of inner ear capillaries (21). Moore et al. (23) reported 12.3% of pediatric cancer patients developed delayed high-frequency hearing loss after long-term methotrexate use, linked to folate metabolism interference and hair cell mitochondrial dysfunction, these changes may facilitate the invasion of immune complexes and inflammatory factors into the inner ear. Furthermore, methotrexate may disrupt folic acid metabolism and energy synthesis in hair cell mitochondria by inhibiting dihydrofolate reductase (23). Additionally, it may suppress the activity of enzymes associated with the tricarboxylic acid cycle, leading to excessive production of reactive oxygen species. This excess can cause mitochondrial dysfunction and subsequently trigger hair cell apoptosis (24).

Previous studies have confirmed that an increase in TNF-*α* secretion can activate the PI3K/AKT/MMP-9 signaling pathway in pericytes, leading to an increase in the permeability of the BLB (Blood Labyrinth Barrier) and ultimately causing hearing impairment. Adalimumab exerts its therapeutic effect by specifically binding to and inhibiting the expression of TNF-α. It is hypothesized that the mechanism causing the aggravation of hearing loss mainly targets the effect of TNFα in AS patients (20). Specifically, as a specific TNFα receptor blocker, adalimumab non-selectively binds to all TNFα pools in the inner ear, including physiologically expressed low-level TNFα, thereby abrogating its interaction with TNFα receptor 1 (TNFR1) and TNFα receptor 2 (TNFR2) (21, 25). This disruption further impairs the normal protective and regulatory functions of low-level TNFα in the inner ear, which mainly include impairment of blood-labyrinth barrier integrity and induction of inner ear hair cell apoptosis (26, 27). According to our research findings, we hypothesize that the two drugs may work synergistically by compromising the blood-labyrinth barrier and disrupting distinct pathways within hair cell mitochondria. The resultant “dual-channel injury” may significantly elevate the risk of sudden deafness (28).

### 4.4 Clinical value

Based on the findings of this study, high-risk patients with a score of ≥8 are recommended to undergo enhanced hearing monitoring during the therapeutic course, receive risk warning references, and participate in quarterly follow-ups. This system can aid clinicians in stratifying patient risk prior to treatment, facilitating closer hearing monitoring for high-risk individuals, and dynamically adjusting medication plans. Table 6 shows further details.

**Table 6:** Hearing monitoring and intervention strategy based on ototoxicity risk stratification.

Risk stratification	Monitoring frequency	Intervention measures
High risk (score ≥ 8)	Pure-tone audiometry monthly	Consider dose reduction or switching to a non-TNF inhibitor
Intermediate risk (score 4–7)	Examination every 3 months	Optimize the control of underlying diseases
Low risk (score ≤ 3)	Routine follow-up	Educate patients on early warning symptoms of tinnitus

### 4.5 Limitations

This study is constrained by the relatively small sample size from a single center, particularly within the case group. The results of the analysis may be limited by both the sample size and potential confounding factors among the patients, including underlying diseases and environmental exposures. These limitations may hinder the depth of exploration into innovative mechanisms. This study did not collect data on patients’ genetic susceptibility, long-term noise exposure history, or history of other ototoxic drug use, which may lead to residual confounding; in addition, the retrospective design cannot completely avoid information bias (e.g., incomplete medical record documentation). Future research should include large sample sizes from multiple centers to further validate the dual-channel hypothesis.

## 5 Conclusion

This retrospective cohort study identified several independent risk factors for sudden sensorineural hearing loss (SSNHL) in patients with ankylosing spondylitis (AS) treated with a combination of adalimumab and methotrexate. These factors include high cumulative drug exposure, prolonged treatment duration, elevated disease activity (as indicated by BASDAI scores and increased CRP levels), and a history of otologic conditions. Notably, the risk profiles vary across hearing subtypes. Total deafness correlates with high drug exposure and significant inflammation, whereas low-frequency decline is primarily associated with tinnitus. The combination therapy is hypothesized to induce SSNHL through a “dual-channel injury” mechanism, wherein adalimumab potentially disrupts the blood-labyrinth barrier, and methotrexate impairs mitochondrial metabolism in hair cells. Consequently, elevated exposure to this drug combination is linked to the onset of SSNHL in AS patients, necessitating heightened awareness of potential risks. Clinically, auditory monitoring should be enhanced for high-risk individuals, and medication regimens should be individualized accordingly. In the future, prospective cohort studies should be conducted to validate the association between the drug regimen and SSNHL, as well as basic experiments to assess the impact of combined therapy on tight junction proteins of the blood-labyrinth barrier and mitochondrial function. Additionally, animal models should be employed to confirm the “dual-channel injury” mechanism.

## Data Availability

The raw data supporting the conclusions of this article will be made available by the authors, without undue reservation.
